# Comparative dissolution, uptake, and toxicity of zinc oxide particles in individual aquatic species and mixed populations

**DOI:** 10.1002/etc.4349

**Published:** 2019-02-18

**Authors:** Fan Wu, Bryan J. Harper, Stacey L. Harper

**Affiliations:** ^1^ School of Chemical, Biological, and Environmental Engineering Oregon State University Corvallis Oregon USA; ^2^ Department of Environmental and Molecular Toxicology Oregon State University Corvallis Oregon USA; ^3^ Oregon Nanoscience and Microtechnologies Institute Eugene Oregon USA

**Keywords:** Nanoparticle, Algae, Aquatic toxicity, *Daphnia*, Zebrafish, Microcosm

## Abstract

Potential differences in species susceptibility to nanoparticle (NP) contaminants make the use of multispecies community toxicity testing strategies beneficial in understanding NP risk to aquatic environments. Because of the limited knowledge of zinc oxide (ZnO) NP fate and toxicity, we conducted multispecies exposures and compared the responses of individual species to the same species in a community comprised of algae (*Chlamydomonas reinhardtii*), bacteria (*Escherichia coli*), crustaceans (*Daphnia magna*), and zebrafish (*Danio rerio*). Different‐sized ZnO particles and ionic Zn were compared to investigate the contribution of particulate and dissolved Zn to aquatic organism toxicity. Each organism and community was exposed to Zn sources at 0.08, 0.8, and 8 mg Zn/L. The present results indicate that all 3 types of Zn elicited differential toxicity among test organisms, with stronger adverse outcomes observed in single species than within a community. The community assay (nanocosm) we developed increased resilience to all Zn exposures by 5 to 10% compared to individual exposures at equivalent concentrations. In addition, the uptake and toxicity of ZnO particles to aquatic communities appear to be driven by rapid dissolution and the concomitant impacts of zinc ion toxicity, and the size of the ZnO particles had little impact on uptake or toxicity. The nanocosm assay could be a useful screening tool for rapidly assessing the potential impacts of nanomaterials to aquatic species. *Environ Toxicol Chem* 2019;38:591–602. © 2019 The Authors. *Environmental Toxicology and Chemistry* published by Wiley Periodicals, Inc. on behalf of SETAC.

## INTRODUCTION

Zinc oxide nanoparticles (ZnO NPs) have the third highest production rates of NPs worldwide (estimates range from 550 to 10 000 tons/yr) because of their extensive use in cosmetics, solar cells, pigments, electronics, and textiles (Wang 2004; Aschberger et al. 2011; Piccinno et al. 2012). The rapid development of the Zn NP industry has arisen from the diverse engineering capabilities which allow for changes in the shape (Hsiao and Huang 2011), size (Hanley et al. 2009), crystallinity (Hariharan 2006), and surface chemistry (Khrenov et al. 2005) of ZnO NPs to support a myriad of uses. Their widespread use increases the risk that ZnO NPs will enter aquatic environments through wastewater streams (Nowack and Bucheli 2007; Scown et al. 2010; Keller and Lazareva 2013). Modeled estimates of ZnO NP environmental concentrations range from 0.001 to 0.058 μg/L in surface waters, from 0.24 to 0.661 μg/kg in soil, and from 0.22 to 1.42 μg/L in sewage treatment plant effluent, where the concentrations are expected to continuously increase (Gottschalk et al. 2009). The likely increases in both the diversity and quantity of ZnO NPs entering the environment necessitate further study of their ecological impacts.

Zinc oxide NPs have been classified as “extremely toxic” (50% lethal effect concentration [LC50] <0.1 mg/L) to aquatic organisms (Kahru and Dubourguier 2010); however, the toxicity to aquatic organisms belonging to various trophic levels varies considerably (Adams et al. 2006; Brayner et al. 2006; Zhang et al. 2007; Franklin et al. 2007; Heinlaan et al. 2008; Huang et al. 2008; Aruoja et al. 2009; Blinova et al. 2010; Wong et al. 2010; Xiong et al. 2011). According to a review by Bondarenko et al. (2013), the reported LC50 values of ZnO NPs for bacteria (multiple species), algae (multiple species), crustaceans (*Daphnia magna*), and fish (*Danio rerio*) varied from 50 to 1000, from 0.052 to 4.56, from 0.62 to 22, and from 1.79 to 4.92 mg/L, respectively. The high variation in toxicological responses is likely related to differences in susceptibility among the test species and variations in experimental conditions such as pH, media composition, temperature, timing and duration of exposure, and other factors.

Zinc oxide NP toxicity has been demonstrated to frequently relate to toxic mechanisms associated with the dissolution and release of ionic Zn (Bacchetta et al. 2016). The generation of reactive oxygen species (ROS) by ZnO NPs has also been shown to induce DNA damage in studies with zebrafish (Bai et al. 2010; Zhao et al. 2013). In addition, the small size of nanomaterials can potentially influence their uptake by organisms (Gliga et al. 2014), as well as their dissolution behavior in aquatic system (Lopes et al. 2014; Abdelmonem et al. 2015) with concomitant impacts on their toxicity relative to their bulk counterparts (Nair et al. 2009; Sharma et al. 2012; Kononenko et al. 2017). As such, understanding how particle dissolution is impacted by ZnO particle size and community structure and the associated impacts on Zn uptake by various organisms is critical to understanding the potential environmental risks.

Studies of ZnO toxicity to aquatic organisms are frequently conducted as laboratory exposures with individual organisms. Although individual species–based ZnO NP toxicity data provide baseline information for assessing the potential risks of ZnO NPs, multispecies toxicity tests can assess the potential impacts of toxicants across trophic levels, identify any indirect effects caused by species interactions (Mackay et al. 1989; Boxall et al. 2002), and capture the variability of exposure routes and the range of sensitivities among organisms (De Zwart and Posthuma 2005). Given the complex nature of ecosystems, it is expected that multispecies toxicity tests would provide an estimation of species‐ and population‐level responses to the toxicant.

Despite the recognition that the sensitivity of organisms to NP contaminants can be altered in the presence of other species (Levy et al. 2009), knowledge of the impacts of engineered NPs to aquatic ecosystems as a whole is still quite limited (Bernhardt et al. 2010). Few investigations have been conducted at environmentally relevant concentrations of ZnO NPs utilizing a microcosm experimental design. One such study in soil microcosms showed that low concentrations of ZnO NPs caused adverse effects on soybean growth (Yoon et al. 2014); however, to our knowledge, no study has investigated environmental impacts of ZnO NPs to various trophic levels of freshwater organisms.

Aquatic microcosm assays have not been commonly applied to nanomaterials because they are costly and time‐consuming, require large quantities of nanomaterials, and generate large amounts of waste (ASTM International 2011). To overcome these barriers, our approach was to develop a rapid, small‐volume multispecies assessment (hereafter referred to as the “nanocosm assay”) that requires minimal amounts of test nanomaterials. In the present study, we compared the responses of individual species including algae (*Chlamydomonas reinhardtii*), bacteria (*Escherichia coli*), invertebrates (*D. magna*), and developing vertebrates (*D. rerio*) to the responses of mixed‐species exposures containing the same organisms to determine the biological impacts of ZnO particles in a community setting. Test species were selected because they represent a broad spectrum of trophic levels and are among the most commonly used in aquatic toxicity testing, particularly for NPs (Supplemental Data, Table S1; Sondi and Salopek‐Sondi 2004; Adams et al. 2006; Harper et al. 2008; Heinlaan et al. 2008; Navarro et al. 2008; Perreault et al. 2012; Chen et al. 2012; Colman et al. 2013; Wu et al. 2017). Multiple endpoints were used to evaluate the impacts of ZnO particle uptake and toxicity at concentrations representing the highest environmentally relevant concentration (0.08 mg Zn/L), an extreme accidental spill scenario (0.8 mg Zn/L), and a much higher concentration (8 mg Zn/L) where adverse impacts could be expected. The long‐term goal of our research group is to establish a validated method for screening nanomaterial ecological risks. The present study focuses on establishing a baseline to compare how the organismal response would differ when exposed to NPs individually or within a community. In the present study, commercially available ZnO NPs, ZnO microparticles (ZnO MPs), and ionic zinc from ZnCl_2_ were selected to investigate the influence of dissolved Zn and particle size on aquatic toxicity to both individuals and communities of aquatic organisms. We hypothesized that the toxicity of ZnO NPs would vary between single and community exposures as a result of differential species susceptibility and interspecies interactions and that dissolved Zn concentrations would dictate the toxicity of the ZnO particles.

## MATERIALS AND METHODS

### Exposure media and organism maintenance

The exposure medium, hereafter referred to as the “nanocosm” medium (NCM; Wu et al. 2017), was prepared by mixing 50% of Taub's 36 solution (Taub and Dollar 1968) with 50% of 5 mM 4‐(2‐hydroxyethyl)‐1‐piperazineethanesulfonic acid (HEPES) buffer (EMD Chemicals), ensuring that the pH of the NCM was maintained at 7.2 before use. Taub medium was selected to minimize NP aggregation because of its low ionic strength and to optimize the growth of algae and daphnids. The HEPES buffer was used as a biological buffer to maintain pH consistent with all of the organisms’ optimal growth. The NCM was stored at 4 °C, autoclaved at 120 °C for 20 min, and cooled to room temperature prior to use.

### Test organisms

Reasons for organism selection are described in detail in the Supplemental Data (Table S1) and our previous study (Wu et al. 2017). *Chlamydomonas reinhardtii* was purchased from the University of Texas Culture Collection (UTEX 2243 and 2244) and cultured in tris‐acetate phosphate (TAP) medium (Gorman and Levine 1965). *Escherichia coli* was purchased from Carolina Biological Supply (MicroKwik culture) and cultured in lysogeny broth medium (consisted of 10 mg/L tryptone, 5 mg/L yeast extract, and 10 mg/L sodium chloride) on a shaker at 37 °C. *Daphnia magna* were maintained in reconstituted moderately hard water consisting of final salt concentrations of 111 mg/L CaSO_4_, 42.65 mg/L MgSO_4_, 117.6 mg/L NaHCO_3_, and 6 mg/L KCl in reverse osmosis water and fed dry spirulina daily. The pH was maintained in the range of 7.8 ± 0.2. Adult wild‐type zebrafish were maintained at the Sinnhuber Aquatic Research Laboratory at Oregon State University. Embryos were collected from group spawns and staged to ensure that all embryos were 6 to 8 h postfertilization at the start of the experiment (Kimmel et al. 1995). All of the organisms were maintained at room temperature of 20.5 ± 0.5 °C with a 16:8‐h light:dark photoperiod under 1690 ± 246 lux light intensity provided by full‐spectrum growth lights.

### Experimental design

Zinc oxide NPs (average primary particle size <50 nm, BET >10.8 m^2^/g), ZnO MPs (<5 μm particle size), and ZnCl_2_ were all purchased in powder form from Sigma‐Aldrich. Zinc oxide particle stock suspensions were prepared at a concentration of 800 mg Zn/L in NCM and sonicated for 2 min at 40% intensity with a VCX 750 Vibra‐Cell sonicator (Sonics & Materials) equipped with a cup‐horn probe and recirculating water bath to maintain temperature. Similar particle stock suspensions were also prepared in Milli‐Q ultrapure water (MQW; EMD Millipore) to investigate the impact of the NCM on particle dissolution and agglomeration. Stock solutions of ZnCl_2_ were also prepared in NCM with equivalent amounts of Zn to the particle suspensions (800 mg Zn/L). Zinc oxide NP suspensions were further diluted to 8 mg Zn/L to facilitate characterization of hydrodynamic diameter (HDD) and zeta potential (ZP) by dynamic light scattering (DLS) using a Zetasizer Nano ZS (Malvern Instruments), with measurements taken every 24 h for 5 d. Detailed parameters for ZP measurements are provided in Supplemental Data, Table S2.

*Chlamydomonas reinhardtii* cells grown in TAP medium were collected during periods of steady growth and centrifuged at 600 *g* for 5 min to pellet the cells. Following removal of the supernatant, the algal cells were resuspended in NCM. For individual and mixed‐species toxicity tests with algae, *C. reinhardtii* cells were inoculated into 50‐mL Falcon® vented tissue culture flasks (Fisher Scientific) at a starting density of approximately 2 × 10^4^ cells mL^−1^ and acclimated for 1 d before starting the experiment. Vented caps were chosen to equalize air pressure inside and outside the container and to prevent contamination from airborne particles and microbes. The total volume of each flask was adjusted to 15 mL, leaving approximately 35 cc of headspace. For tests incorporating bacteria, *E. coli* inoculates were added to the 50‐mL flasks to provide a final density of 5 × 10^5^ cells mL^−1^. Initial algal and bacterial cell densities were quantified using an Accuri C6 flow cytometer (BD Biosciences) to ensure consistency in our initial cell densities at the start of the experiment. *Daphnia magna* neonates (<24 h old) were collected from the stock culture and acclimated in NCM for 24 h prior to toxicity testing. Zebrafish embryos were acclimated in NCM for 3 h prior to testing. For *D. magna* and zebrafish exposures (alone or in conjunction with algae and bacteria), 5 neonate *D. magna* and 8 zebrafish embryos were introduced into each 50‐mL flask. Five replicate flasks were prepared for each exposure scenario at 0, 0.08, 0.8, and 8 mg Zn/L for each type of Zn exposure (including ZnCl_2_).

### Toxicity evaluations

Algal and bacterial viability was measured using SYTOX Green dead cell stain (Life Technologies) in conjunction with flow cytometry at 2 and 6 h postexposure for bacteria, then daily for the remainder of the experiment for both algae and bacteria. *Daphnia magna* mortality and immobilization was recorded daily. The *D*. *rerio* embryo hatching rate and mortality were monitored daily. At the end of the 5‐d exposure, zebrafish embryos were examined under a dissecting microscope for morphological malformations (body axis, brain, heart, eyes, fins, jaw, trunk, somite), physiological abnormalities (pigmentation, circulatory system, pericardial edema, yolk sac edema), and altered behaviors (touch response, hatching rate). *Danio rerio* embryo developmental stage was corrected according to Kimmel et al. (1995; Equation (1)) because of the change in experimental temperature

(1) $$H_{T}=h\text{/}(0.055T-0.57)$$

where *H_T_* represents the hours of development at temperature *T* and *h* represents the hours of development to reach that stage at 28.5 °C.

### Measurement of Zn dissolution and organismal uptake

The abiotic rate of dissolution for both sizes of ZnO particles at 8 mg Zn/L was measured in NCM initially and after 1 and 5 d in triplicate. Dissolved Zn concentrations in each exposure scenario (biotic dissolution) were also measured at days 1 and 5 at all exposure concentrations in triplicate (randomly selected from 5 exposure replicates). Nanocosm flasks were gently agitated prior to sampling to resuspend any settled particles at each time point, then a 0.5‐mL aliquot was taken from the flask and centrifuged at 8000 rpm for 10 min through a 3‐kDa centrifugal filter (VWR) to remove undissolved particles. Filtered medium was transferred to a polystyrene tube and acidified with trace metal–grade nitric acid (Fisher Scientific) prior to analysis of Zn content by inductively coupled plasma optical emission spectrometry (ICP‐OES; Teledyne Technologies). A Zn ICP standard was purchased from Sigma‐Aldrich and diluted to concentrations ranging from 0 to 8 mg Zn/L for analysis. The maximum measured dissolved Zn concentration from the exposures determined by ICP‐OES was further used to model the speciation of dissolved Zn using Visual MINTEQ, Ver 3.1.

Zinc uptake by organisms was measured at the end of the experiment following toxicological observations. Daphnids and zebrafish were rinsed 3 times with MQW to remove loosely attached algae, bacteria, and particles. Unhatched zebrafish were dissected to remove the chorionic membrane. Chorionic membranes, zebrafish embryos, and daphnids were stored at −4 °C in polystyrene tubes prior to acid digestion with trace metal–grade nitric acid according to established methods (Wu et al. 2017). After digestion, all samples contained a final proportion of 3% nitric acid and were analyzed for Zn content by ICP‐OES. Three sample replicates were prepared for ICP analysis from each exposure scenario at each exposure concentration.

### Statistical analysis

Algal and bacterial survival was calculated using the Henderson‐Tilton formula (Equation (2)) to compensate for possible differences in the surviving proportion of organisms occurring posttreatment

(2) $$\text{Corrected}\;\text{survival}(\%)=\left(\frac{n_{\text{Cb}}{\times n}_{\text{Ta}}}{n_{\text{Ca}}{\times n}_{\text{Tb}}}\right)\times \text{100}$$

where *n_C_* is the survival proportion (live cell/total cell) in the control group, *n_T_* is the survival proportion in the treatment group, and *a* and *b* designate after and before treatment, respectively (Henderson and Tilton 1955). The intrinsic growth rates of algae and bacteria were calculated using a 3‐parameter logistic model (Equation (3))

(3) $$M=\frac{k}{\left[\text{1+}\left(\frac{k}{M0-1}\right)\right]\times e^{-rt}}$$

where *r* is the specific growth rate (per hour), *t* is the time (hours), *k* represents the maximum capacity of cells, *M_0_* is the initial cell count at time (*t*) = 0, and *M* represents the cell count at *t* = *t* (Paine et al. 2012). SigmaPlot (Systat Software) was used to statistically analyze changes in algal and bacterial survival over time using the Kruskal‐Wallis rank sum test in conjunction with a Tukey's post hoc analysis. Zinc dissolution and uptake concentrations were each compared using one‐way analysis of variance (ANOVA) at different time points. Growth rates of algae and bacteria were calculated in SAS 9.3 (SAS Institute) using a sigmoid growth model (Gauss‐Newton method). *Daphnia magna* survival in control and treatments was compared using a 2‐way ANOVA with a Tukey post hoc test. Differences in the frequency of developmental abnormalities in *D. rerio* between treatments and the corresponding control were analyzed with Fisher's exact test. The integrated change in survival for all organisms (Δ_surv_) was calculated as the difference between the community and individual exposure responses. Differences were considered statistically significant at *p* ≤ 0.05 for all analyses.

## RESULTS

### NP suspension characterization

**Figure 1:**
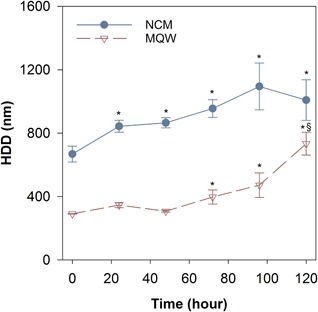
Hydrodynamic diameter of ZnO nanoparticles (8 mg Zn/L) in nanocosm medium (NCM) and Milli‐Q water (MQW) over 120 h. Solid line with filled circles represents responses measured in NCM, and dashed line with open triangles represents the responses measured in MQW. Error bars correspond to the standard error for each set of measurements. *Significant difference compared to corresponding initial measurements; ^§^significant difference from previous time point. HDD = hydrodynamic diameter.

Nanoparticle agglomeration occurred in both the experimental NCM and MQW as evidenced by the HDD of the ZnO NPs at 8 mg Zn/L measured by DLS (Figure 1). The initial HDD of ZnO NPs suspended in MQW was 291 ± 2 nm. In contrast, the HDD of the ZnO NPs suspended in the NCM was much larger (668 ± 51 nm). Zinc oxide NP agglomeration increased significantly at 72 h in the MQW, reaching a final HDD of 734 ± 72 nm after 120 h. Significantly larger agglomerates were observed in the NCM with the HDD increased to 1008 ± 128 nm after 120 h. The ZP for the NPs in NCM did not differ significantly over the 5‐d experimental period (–15.7 to –16.9 mV), suggesting that the majority of particle agglomeration occurred almost instantaneously (Supplemental Data, Figure S1). The ZP was not determined in the MQW because of the lack of conductivity for determining electrophoretic mobility (Lowry et al. 2016).

### NP dissolution

#### Abiotic dissolution

**Figure 2:**
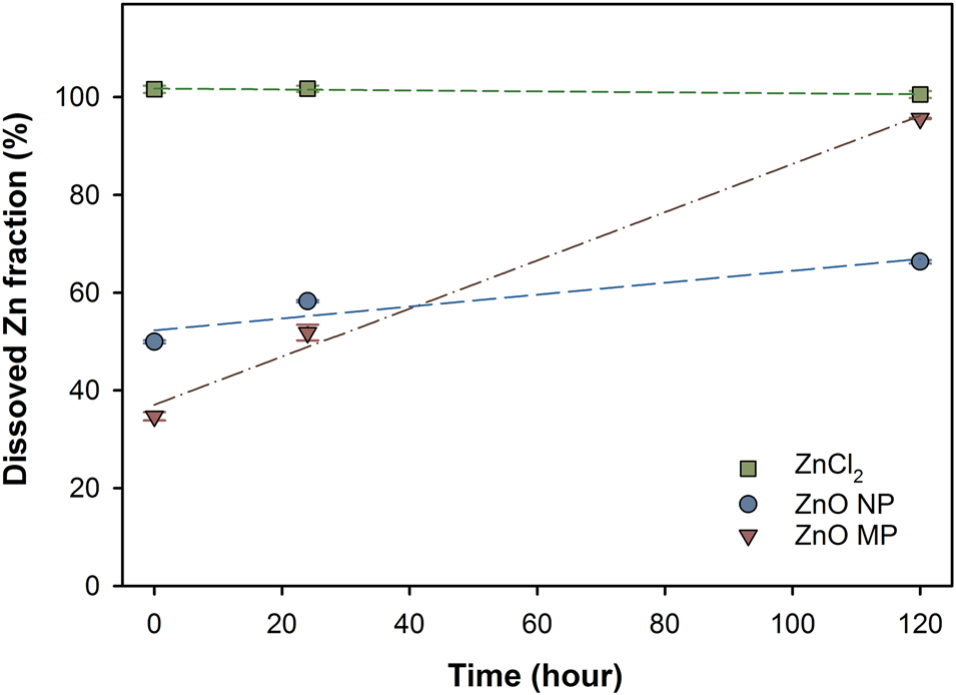
Dissolved Zn concentration for all 3 types of Zn exposures (normalized to 8 mg Zn/L) measured in nanocosm medium over 120 h. Error bars represent the standard error derived for triplicate samples. Dashed lines show linear regressions used for dissolution rate calculations. MP = microparticle; NP = nanoparticle.

The dissolved Zn concentrations resulting from each type of Zn in the NCM are presented in Figure 2. Zinc chloride had near instantaneous and complete dissolution, whereas the ZnO NPs and MPs had initial dissolved Zn concentrations representing 50 and 35% of the initial concentration, respectively. After 24 h, there was a significant increase in the dissolved Zn concentration for both ZnO NPs and MPs. The final 120‐h dissolved Zn concentration was significantly higher for the ZnO MPs, with nearly 95% of the particles dissolved, relative to 66% of the total concentration for the NPs. Overall the ZnO MPs had a higher dissolution rate over 5 d (0.49 ± 0.04% Zn/h) compared to ZnO NPs (0.12 ± 0.04% Zn/h), contributing to the significantly higher final dissolved Zn concentration observed for the MPs (Figure 2).

#### Biotic dissolution

**Figure 3:**
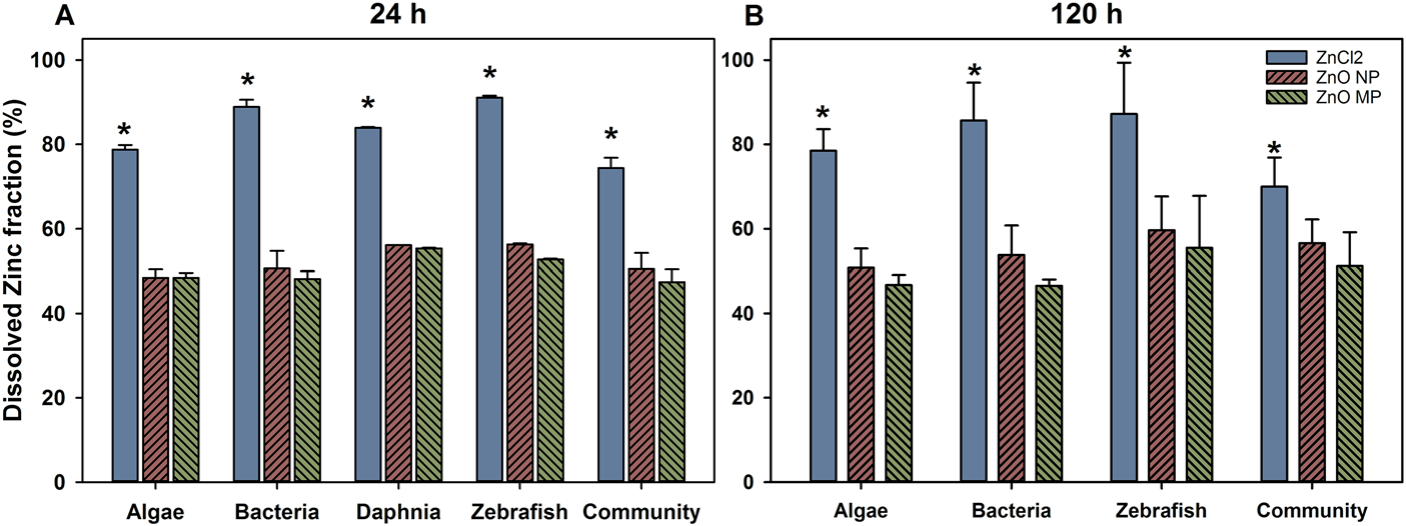
Dissolved Zn fraction measured in nanocosm medium following addition of 0.8 mg Zn/L after 24 (**A**) and 120 (**B**) h for each exposure scenario (*Daphnia* at 120 h were not measured because of limited survival after 48 h). Error bars represent the standard error derived for triplicate samples. *Significant difference between Zn types. MP = microparticle; NP = nanoparticle.

Dissolved Zn in experimental flasks representing each level of organismal complexity was measured at 24 and 120 h for all 3 types of Zn exposures. When organisms were present, the amount of dissolved Zn in the ZnCl_2_ exposures was in the range of 70 to 87% of the initial amount added for the 8 mg Zn/L exposure (Figure 3). Although the presence of organisms decreased the dissolved Zn fraction, ZnCl_2_ still had significantly more dissolved Zn than was found for either particle exposure. In addition, there was no significant difference in the amount of dissolved Zn present at 24 and 120 h across all exposure scenarios.

#### Dissolved Zn speciation

The chemical equilibrium model Visual MINTEQ V3.1 was employed to investigate the speciation of released Zn ions within the NCM. By assuming that all of the Zn added in each experiment had dissolved, we find that, according to the model, even at 8 mg Zn/L, the majority of the Zn remained present as dissolved Zn (86% as Zn^2+^) and that Zn precipitates were minimized (Supplemental Data, Table S3). The modeling results also suggest that when there is less Zn added to the NCM (i.e., 0.08 mg Zn/L), most of the dissolved species would complex with ethylenediaminetetraacetic acid in the medium rather than being present as free Zn^2+^ in solution.

### Zn uptake

**Figure 4:**
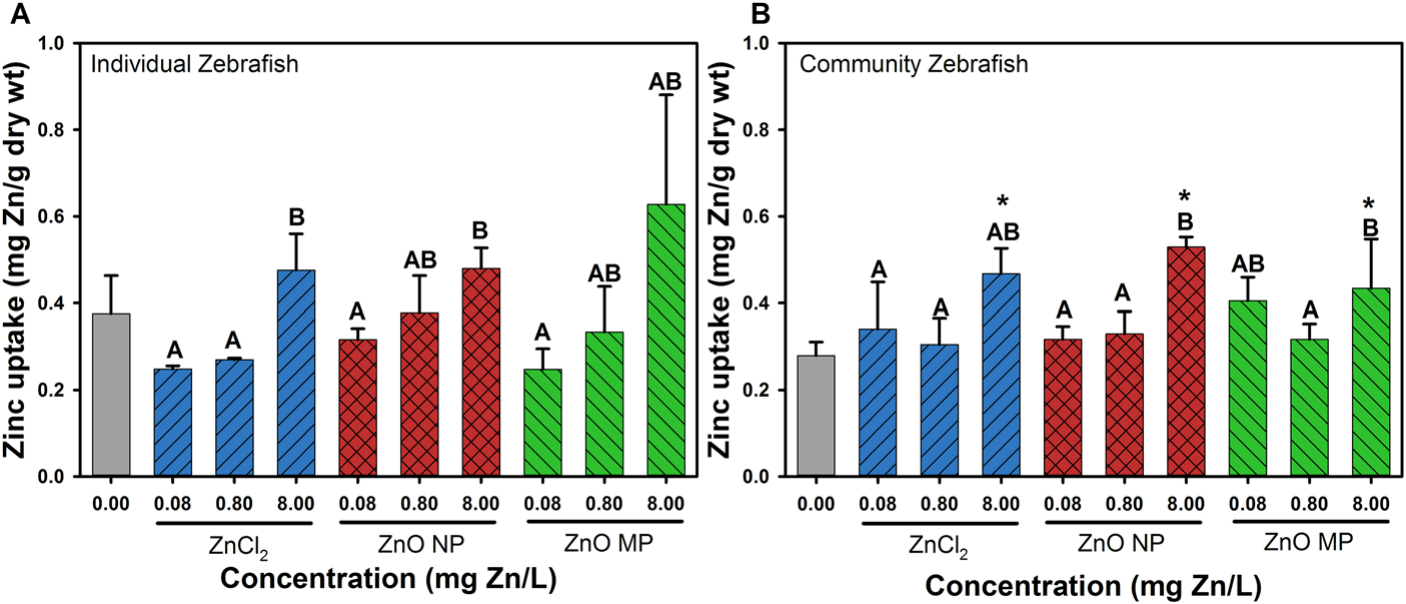
Zebrafish Zn uptake after 120‐h exposure to 0.08, 0.8, and 8 mg Zn/L in individual species (**A**) and community (**B**) exposure scenarios. Error bars represent the standard error derived from 5 sample replicates. Uppercase letters indicate significant difference among exposure types at varying concentrations. *Significant difference in uptake relative to control zebrafish. MP = microparticle; NP = nanoparticle.

Zinc uptake in *D. magna* and zebrafish embryos following 120 h of exposure to each of the 3 types of Zn is shown in Supplemental Data, Figure S2 and Figure 4, respectively. *Daphnia magna* Zn uptake was only measured at 120 h in the community flasks at exposure concentrations of 0, 0.08, and 0.8 mg Zn/L because of the 100% mortality that occurred at 8 mg/L after 48 h of exposure (Supplemental Data, Figures S2–S4). There was a concentration‐dependent increase in Zn uptake in *D. magna* following all 3 types of Zn exposure. Following exposure to 0.08 mg Zn/L as ZnCl_2_, there was significantly higher Zn uptake in *D. magna* than was found in ZnO NP and MP exposures; however, there was no significant difference in uptake among the 3 different types of Zn exposures at 0.8 mg Zn/L (Supplemental Data, Figure S2).

Zebrafish Zn uptake in single‐species and community exposures showed concentration‐dependent uptake of all forms of Zn (Figure 4). A significantly higher Zn concentration was measured in zebrafish exposed to 8 mg Zn/L from any Zn type in the community exposures than was found in control embryos; however, this was not found in the individual species exposures.

### Organism toxicity

#### Algal responses

**Figure 5:**
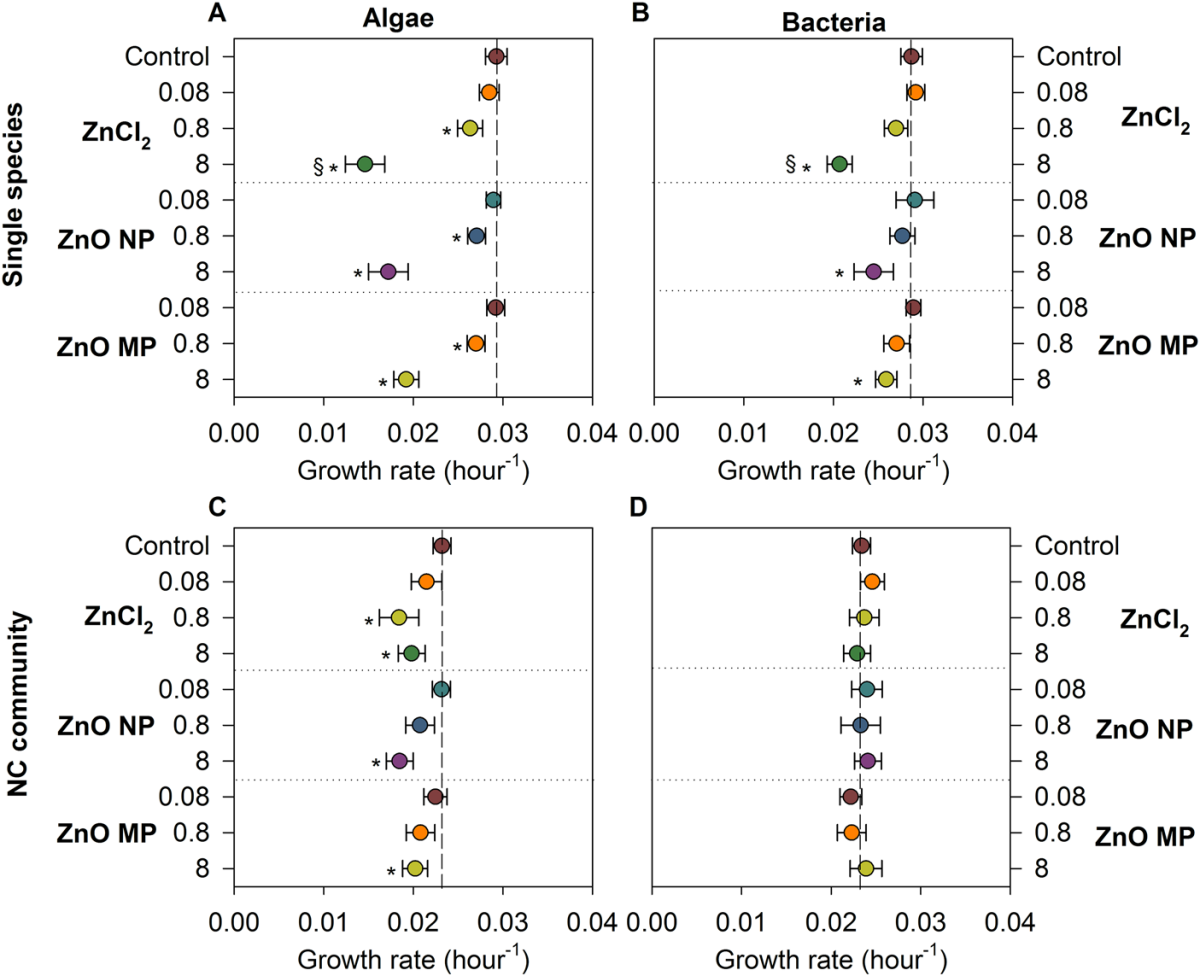
Growth rates of algae (left panel) and bacteria (right panel) in different exposure scenarios: single species (**A**, **C**), and all 4 species combined in (**B**, **D**). Vertical reference lines indicate control growth rates, and horizontal reference lines separate the type of Zn exposures. Error bars represent the standard error derived from 5 sample replicates. *Significant difference from the corresponding control; ^§^significantly lower growth rate among the 3 Zn exposures at the same concentration. MP = microparticle; NC = nanocosm; NP = nanoparticle.

All 3 types of Zn exposures showed a significant concentration‐dependent impact on algal growth rates regardless of the number of species present (Figure 5A, B). The growth rate of algae in the individual‐species exposures was significantly decreased compared to control growth rates (0.029 h^−1^) by all types of Zn exposures at concentrations ≥0.8 mg Zn/L (Figure 5A). The highest ZnCl_2_ exposure (8 mg Zn/L) elicited significant decreases in algal growth rate relative to particulate exposures (NPs or MPs) at the same Zn concentration in the single‐species exposure; however, this difference was not found in the community exposure. In the community exposure, significant algal growth inhibition relative to control (0.023 h^−1^) was observed at 0.8 mg Zn/L in the ZnCl_2_ exposure, yet significant growth inhibition only occurred at the highest concentration (8 mg Zn/L) in both particulate exposures.

#### Bacterial responses

The control growth rate for bacteria in the individual‐species exposure was found to be 0.029 h^−1^, whereas in the community exposure the control growth rate was slightly lower (0.023 h^−1^). In individual species bacterial exposures, ZnCl_2_ at 8 mg Zn/L caused significant growth rate inhibition relative to the rates for both particulate exposures at the same Zn concentration. In addition, 8 mg Zn/L in particulate exposures resulted in significant reduction in bacterial growth rates relative to control, with no effect on growth rate noted at any of the lower exposure concentrations. In the nanocosm community exposure, there was no significant difference in bacterial growth rates with any type of Zn at any exposure concentration.

#### Daphnia responses

The differences in toxicity of Zn materials to *D. magna* following individual and community exposures were compared at 48 h because of the limitation of nutrients and food to support daphnid growth in the individual exposures beyond this point (Supplemental Data, Figure S3). In both the single‐species and community exposure scenarios, no significant mortality to *D. magna* was observed for any type of Zn exposure at the 2 lowest exposure concentrations (0.08 and 0.8 mg Zn/L), whereas 100% mortality was observed at 8 mg Zn/L for all types of Zn in the individual‐species exposure (Supplemental Data, Figure S3a) and significant decreases in survival relative to control occurred in the community exposure. In the single‐species exposures, ZnCl_2_ elicited higher mortality compared to both particle exposures at 24 h, whereas significantly higher mortality than particulate exposures was only found for ZnCl_2_ in the community exposures after 48 h (Supplemental Data, Figure S3b).

#### Zebrafish responses

**Figure 6:**
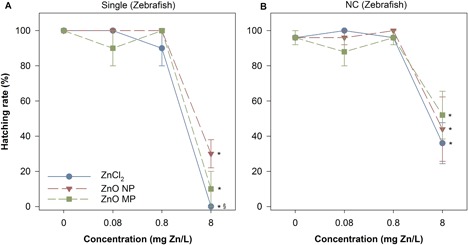
Zebrafish hatching rate after 120 h in single (**a**) and NC (**b**) exposures to single (**a**) and community (**b**) exposure conditions. *Significant difference from corresponding control; ^§^significant difference between ZnCl_2_ and particulate exposures. Error bars represent the standard error derived from 5 sample replicates. MP = microparticle; NC = nanocosm; NP = nanoparticle.

There was no significant difference in mortality or sublethal malformations at exposures up to 8 mg Zn/L, regardless of the type of Zn in either the single‐species or the community scenario. There were significant impacts on embryo hatching rate over the 120‐h exposure, with significantly delayed hatching occurring at 8 mg Zn/L in all Zn exposures for both single‐species and community exposure scenarios (Figure 6). Zinc chloride led to a significantly higher percentage of embryos experiencing delayed hatching than either of the particle exposures in the single‐species exposures, yet this difference did not occur in the community exposure scenario.

### Integrated comparison

**Figure 7:**
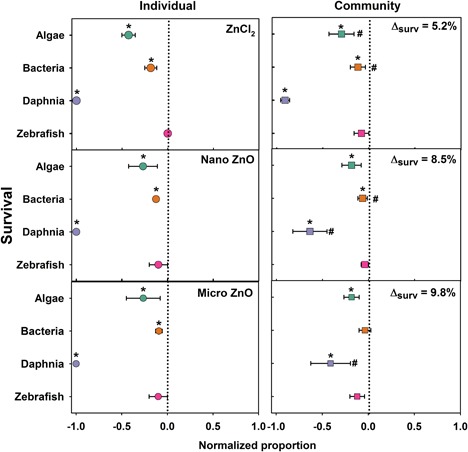
The overall 120‐h survival comparison for each organism in single‐species and nanocosm community exposed to 8 mg Zn/L. Dashed line represents the normalized individual species and community control responses. Error bars represent the standard error derived from 5 sample replicates. *Significant difference compared to corresponding control; ^#^significant difference between individual species and community exposures. The mean toxicity shift for all 4 species in the nanocosm community compared to the single‐exposure scenario is represented by Δ_surv_.

To compare the toxicity across all species in each exposure scenario, we compiled an integrated comparison of toxicity for the species. We normalized the survival proportions for each organism using Equation (2) to compare between individual species and community exposures (Figure 7). The corresponding control of each species was set to 0 (dashed lines in the center of each panel), such that the deviation from control in Figure 7 indicates positive (>0) or negative (<0) impacts on organism 120‐h survival to Zn exposure at the highest test concentration (8 mg Zn/L).

At the lower exposure concentrations (0.08 and 0.8 mg Zn/L), deviations in organismal toxicity were not significantly different from control survival in single‐species or community exposures (Supplemental Data, Figures S5 and S6). However, at 8 mg Zn/L there was a significant reduction in toxicity in the community exposure at 8 mg Zn/L compared to single‐species exposures. By comparing the deviation of each organism in nanocosm to single exposure, the overall resilience (ability to resist impact) was calculated as the percentage deviation of species response in the nanocosm to single species. The resilience of community exposure was increased by 8.5, 9.8, and 5.2% for ZnO NP, MP, and ZnCl_2_, respectively, for organism survival.

## DISCUSSION

### Exposure characterization

The large HDD of ZnO NPs in NCM indicates that a significant agglomeration of ZnO NPs occurred over the experimental period, with agglomeration being highest in the NCM relative to MQW (Figure 1). The increased agglomeration in the NCM can likely be attributed to the presence of various cations in the NCM, which compress the electrical double layer and increase the likelihood of particle–particle interactions (Prathna et al. 2011). Divalent cations in particular, such as those in our NCM, have been demonstrated to impact the stability of NPs in aqueous media (Baalousha et al. 2013; Miao et al. 2016). Large agglomerates also formed in MQW, suggesting instability of the ZnO NPs in suspension, even in pure water.

Typically, ZP would be used to measure suspension stability; however, the low conductivity of pure water precludes accurate measurement (Lowry et al. 2016). The ZP in the NCM was slightly negative and remained fairly constant (–15.7 to –16.9 mV) over 5 d in solution, further supporting the lack of suspension stability leading to agglomeration. The pH of our NCM was buffered at 7.2, which may have contributed to some of the agglomeration. However, the point of zero charge for ZnO NPs is known to be at a pH range of 8.7 to 10.3 depending on medium composition (Kosmulski 2001), with most studies showing pH 9 as the closest to the point of zero charge for ZnO NPs (Bian et al. 2011; Omar et al. 2014). The significant agglomeration resulted in particle agglomerates approaching the micron scale after 72 h but remaining much smaller than the MPs used in the study. It should be noted that once the particles are introduced to a biotic environment, there is the possibility that biological exudates could coat the NPs and stabilize the particle suspension, resulting in smaller HDDs than were measured in an abiotic environment (Zhou and Keller 2010; Louie et al. 2016).

In addition to the nearly complete dissolution of the ZnCl_2_ in the NCM, both sizes of ZnO particles had rapid initial dissolution in the NCM, which resulted in high dissolved Zn concentrations (Figure 2). The rapid dissolution measured in the NCM is similar to the dissolution kinetics previously reported for ZnO in other types of suspension media (Xia et al. 2008; Adam et al. 2014; Xiao et al. 2015). The amount of dissolved Zn released from the ZnO NPs was similar to that reported by Merdzan et al. (2014), who found that >85% of Zn ions were released from bare ZnO NPs in HEPES buffer after 24 h. This rapid dissolution suggests that the HDD values measured for the NPs after 120 h may have increased solely because of the dissolution of the smaller agglomerates in the suspension, thus shifting the average diameter measured by DLS toward the larger particles. The combined effects of the NP surface coating (Merdzan et al. 2014), particle size (Meulenkamp 1998), and water parameters of the exposure medium (Li and Wang 2013) have all been reported to critically impact NP dissolution; however, in the present study, only particle size differed, suggesting that size in this case impacted the rate of dissolution. The ZnO NPs in the present study had a more rapid initial release of dissolved Zn than MPs, but MPs had the highest dissolution rate over the experimental period when no organisms were present.

In the biotic dissolution study, the dissolved Zn fractions for the 2 particle types were consistent after 24 h and in the range of the dissolved fractions measured for the NPs in the abiotic dissolution experiments. The lower dissolved Zn in the MP biotic exposures suggests some form of biotic surface passivation of dissolution. Organic matter can coat NPs and MPs and prevent them from further releasing ions (Adeleye et al. 2014). It is also possible that the organisms took up dissolved Zn, lowering the amount measured in solution; however, we would expect this to be constant across all Zn types. The ZnCl_2_ exposures suggest that Zn ions were bound to the organisms or actively taken up as the concentrations measured in solution were at least 10% lower than those in the abiotic dissolution study. The secretion of extracellular organic carbon by *C. reinhardtii* and *E. coli* can coat NPs (Baba et al. 2011; Kadar et al. 2012), significantly reducing dissolution with concomitant impacts on the toxicity of NPs (Stevenson et al. 2013). In addition, metal NP toxicity can be reduced in the presence of dissolved organic carbon (DOC; Blinova et al. 2013); with the potential of more DOC generated in the nanocosm than individual species scenario, toxicity can also be mitigated.

### Zn uptake by organisms

The measured Zn uptake in *D. magna* and zebrafish was not significantly different among the 3 Zn sources at the same exposure concentrations, suggesting that dissolved Zn species were likely the Zn species readily taken up by the organisms. The results of Visual MINTEQ estimations show that >90% of the measured Zn was present as free Zn^2+^, supporting the uptake by organisms being primarily ionic Zn. Comparing the biotic and abiotic dissolved Zn concentrations, the presence of organisms significantly decreased the dissolved Zn concentrations in ZnO NP at 24 h (Figure 3A) and MPs at 120 h (Figure 3b). However, there is no clear trend indicating which organism had the most impact on Zn dissolution. Although all species affected the dissolution of ZnO NPs and MPs, the effects were not additive because the community did not elicit significantly different dissolved Zn compared to other single‐exposure scenarios.

### Zn toxicity

Generally, the mechanisms of ZnO NP toxicity to organisms are attributed to the release of Zn ions, which disrupts homeostasis (Chevallet et al. 2016), the generation of ROS and the subsequent oxidative stress (Zhang et al. 2010), and/or membrane disorganization and internalization resulting from direct particle–cell contact (Adams et al. 2006; Huang et al. 2008). The toxicity to all 4 species is highly correlated with the dissolved Zn fraction, implying that mechanisms associated with dissolved Zn dictated the observed toxicity. In addition, ZnCl_2_ elicited higher toxicity than either the nano‐ or micron‐sized particles, further supporting the notion that dissolved Zn dominated the observed toxicity more than any particle‐specific effect. In fact, no particle‐specific impacts were observed for any organisms in the present study.

The ZnCl_2_ exposures had the largest impact on algal growth rate and survival, although the particulate exposures did not differ in their impacts on either growth rate or survival, providing evidence that soluble species drove algal responses. Studies have shown that algae such as *C. reinhardtii* are more susceptible to soluble species than suspended solid particulates (Gunawan et al. 2013). Zinc oxide NPs contained more smaller, membrane‐permeable particles, which could potentially be internalized by algae and bacteria and cause toxicity (Gunawan et al. 2013). However, the large HDDs at 8 mg Zn/L and comparable toxicity with the MP exposures indicate that the particles themselves were likely not serving as the major toxic agent to the algae. In the community exposures the Zn impacts on algal growth rate were decreased relative to the individual‐species exposures (Figure 5); however, algal survival was only significantly different in the ZnCl_2_ exposures relative to the individual‐species survival (Figure 7).

The ZnCl_2_ exposures had the largest impacts on bacterial growth rates (Figure 5); however, the highest concentrations of particulate Zn also significantly decreased bacterial growth rates. Despite the differences in bacterial growth rates, there was no significant difference between the Zn types on the survival of bacteria during when exposed alone (Figure 7). When exposed as a community, none of the Zn types impacted bacterial growth rates at any concentration and only ZnCl_2_ and ZnO NPs impacted survival. Previous studies have reported *E. coli* having low sensitivity to Zn salt and NP exposures (Bondarenko et al. 2013), but in the present study, ZnO NPs inhibited the growth of *E. coli* only in the individual exposure. The toxicity of ZnO NPs to *E. coli* could vary depending on the composition of the test medium. For instance, CeO_2_ NPs were highly toxic to *E. coli* in pure water (Thill et al. 2006), but no effect on *E. coli* was found in culture media because the CeO_2_ NPs formed large aggregates (Zeyons et al. 2009).

*Daphnia magna* were much more susceptible to Zn exposures than algae or bacteria. Because of their filter‐feeding behavior, it is possible that daphnids were exposed to Zn from suspended particles in the water column and from preying on Zn‐contaminated algae and bacteria; however, particulate exposures did not result in higher levels of internalized Zn (Supplemental Data, Figure S2). Zinc chloride elicited higher toxicity to *D. magna* than ZnO NPs and MPs, likely because of the higher Zn concentration that was measured within the *D. magna* body under ZnCl_2_ exposures. This is consistent with what was observed in previous studies which concluded that the toxicity of ZnO NPs was triggered by the release of ionic Zn from NPs (Ergonul et al. 2012; Bondarenko et al. 2013; Seo et al. 2014).

Significant hatching delay in zebrafish embryos was observed in both individual and community exposures, likely attributable to the high concentrations of dissolved Zn released from ZnO particles (Figure 3). In zebrafish embryos Zn^2+^ is essential for both development and fertilization (Zhao et al. 2014); however, an overabundance of Zn^2+^ has been shown to inhibit the development of embryonic zebrafish, and a high concentration of Zn^2+^ may cause ionic competition, altering their nutrient uptake and eventually disrupting the normal hatching function. Bai and colleagues (2010) reported a concentration‐dependent decrease in hatching rate of zebrafish embryos following exposure to ZnO NPs and suggested that delayed hatching might be caused by the disturbance of the Zn‐protease hatching enzyme by ionic Zn and hypoxia induced by ZnO NPs blocking the channels in the chorionic membrane. Given the large HDD of the ZnO NP agglomerates in the NCM and the primary size of MPs, it is feasible that large agglomerates coated the chorion, which then interfered with embryo oxygen exchange, leading to a decreased hatching rate (Cheng et al. 2007). However, because of the rapid dissolution of the ZnO particles, it is more likely that dissolved Zn species were responsible for the observed delayed hatching. This is further supported by the significantly increased hatching delay in the ZnCl_2_ exposures.

The individual species and nanocosm community exposure scenarios had different limiting factors. In the single‐species exposures, toxicity was elicited directly from the Zn material exposures; however, in the community exposures the effective Zn exposure was impacted by the interactions among species, including trophic transfer and interspecies interaction, therefore altering toxicity. For a given Zn exposure, the community exposures had more biological sinks, which then reduces the effective exposure, providing increased resilience to the system. In contrast is the potential for the organisms to accumulate Zn and transfer that Zn to higher trophic levels or otherwise alter the dissolution and release of dissolved Zn. Only the highest exposure concentration (8 mg Zn/L) elicited significant toxicity to the targeting organisms. Thus, no effect should be expected in natural communities, given that no significant effect is found even for our lowest exposure concentration at 0.08 mg Zn/L, which is approximately 50‐fold higher than the upper bound exposure level at wastewater effluents (0.00142 mg/L). Other exposure scenarios, such as an accidental spill, could significantly raise environmental concentrations but would only cause adverse impacts at an extremely high concentration.

The nanocosm community assay we developed has shown increased resilience to all 3 Zn exposures compared to single‐species exposures. Similar results have been reported for silver NPs, where the toxicities of Ag NP in outdoor mesocosm, microcosm, and conventional laboratory studies were compared and the lowest toxicity was observed in larger‐scale mesocosms (Bone et al. 2015). The nanocosm assay is a rapid and cost‐effective method to screen and evaluate the environmental toxicity of NPs.

## CONCLUSIONS

Overall, the present study demonstrates that dissolved Zn dictates the uptake behavior and toxicity of ZnO particles to aquatic communities through rapid dissolution and the concomitant impacts of Zn ion toxicity. However, current estimated environmental concentrations for ZnO NPs are too low to elicit adverse impacts to the aquatic ecosystems. Lower toxicity and delayed impacts were observed for each species when exposed as a community rather than as individual species, demonstrating the increased resilience to ZnO particles with increased species complexity. The size of the ZnO particles had little impact on the findings, even though the filter‐feeding daphnids were most impacted by the exposures. Future research will focus on validating the nanocosm assay as a rapid, low‐cost approach for assessing the potential environmental impact of NPs across a range of species simultaneously.

## Supplemental Data

The Supplemental Data are available on the Wiley Online Library at DOI: 10.1002/etc.4349.

## Supporting information

This article includes online‐only Supplemental Data.

Supporting Data S1.
