# Supplementary material for: Comparative dissolution, uptake, and toxicity of zinc oxide particles in individual aquatic species and mixed populations
Source: Environ Toxicol Chem. 2019 Feb 18;38(3):591–602. doi: 10.1002/etc.4349 (PMC6446720; doi:10.1002/etc.4349)
Supplement: Supplementary file 1 — Supporting Data S1. [file ETC-38-591-s001.docx]

**Comparative dissolution, uptake and toxicity of zinc oxide particles in individual aquatic species and mixed populations**

*Fan Wu^1^, Bryan J. Harper^2^, Stacey L. Harper^1,2,3*^*

^1^School of Chemical, Biological and Environmental Engineering, Oregon State University, Corvallis, OR, United States; ^2^Department of Environmental and Molecular Toxicology, Oregon State University, Corvallis, OR, United States; ^3^Oregon Nanoscience and Microtechnologies Institute, Eugene, Oregon, United States

**Supplemental Information:**

2 Tables, 6 Figures

***Organism Selection:***

Four species (*Chlamydomonas reinhardtii*, *Escherchia coli, Daphnia magna, Danio rerio)* were selected as test species based on their extensive use in aquatic nanotoxicity tests and represent a broad spectrum of trophic levels ([Sondi and Salopek-Sondi 2004](#_ENREF_230); [Adams, Lyon et al. 2006](#_ENREF_6); [Harper, Usenko et al. 2008](#_ENREF_110); [Heinlaan, Ivask et al. 2008](#_ENREF_113); [Navarro, Piccapietra et al. 2008](#_ENREF_192); [Chen, Zhou et al. 2012](#_ENREF_52); [Perreault, Oukarroum et al. 2012](#_ENREF_205); [Colman, Arnaout et al. 2013](#_ENREF_60)). *C. reinhardtii* and *E. coli* were chosen to represent primary producers and decomposers, respectively. Both species have a simple life cycle, are inexpensive to maintain, are commonly found in aquatic environments and interspecific competition between the two species has been reported ([Levy, Stauber et al. 2009](#_ENREF_157)). While bacteria and plankton compete for resources, both also serve as food for the primary consumer *D. magna*. *D. magna* are small aquatic crustaceans ubiquitous in freshwater lotic environments and were included as a primary grazer of the microorganisms. *D. magna* are sensitive to chemical stressors and have long been utilized as an indicator species for assessing aquatic contamination. Embryonic zebrafish were selected as an ideal developing vertebrate model due to their: (i) rapid development, (ii) relatively high sensitivity to anthropogenic contaminants at embryonic stage, (iii) transparency for visual observations, and (iv) well-studied sub-lethal endpoints elicited from NP exposures ([Harper, Carriere et al. 2011](#_ENREF_111)). Although the embryonic zebrafish do not actively participate in the food web given the short timeframe of these studies, they are exposed to the microcosm contaminants throughout the experiment and following hatching. Mouth-gaping behavior begins around the time of hatching which can lead to oral ingestion of contaminants, despite not actively feeding.

***Table S1****. Organism selection and evaluated endpoints.*

| Organism level | Species name | Reason for selection | Endpoints |
| --- | --- | --- | --- |
| Primary producer | *Chlamydomonas reinhardtii* | Easy to maintain; short life cycle; well-studied; multiple endpoints | Growth rate; cell viability |
| Decomposer | *Escherichia coli* | Well studied in nanotoxicity testing; short life cycle; easy to culture; | Growth rate; cell viability |
| Primary consumer | *Daphnia magna* | Indicator species for assessing aquatic contamination; widely existing in aquatic ecosystems; transparent body allows easily observed endpoints | Survivability; immobilization; uptake |
| Secondary consumer (indirect) | *Danio rerio (embryonic)* | Alternative animal model, rapidly develop, easy for observation | Mortality, developmental progression; malformation; behavior; uptake |

Table S2. Standardized information for determining nanoparticle zeta potential in NCM:

| Surface functionalization | None |
| --- | --- |
| Shape | spherical |
| Model used to compute the zeta potential | Henry's Equation (Smoluchowski approximation) |
| Duration of measurement | 90 seconds for single measurement |
| Applied voltage | 148 V |
| Number of measurements made and averaged to determine each ZP | 12 |
| Total number of replicate measurements | 3 |
| pH | 7.2 ± 0.2 |
| Ionic strength | 0.01 mol/L |
| Ionic composition | Made according to Ref. 47 |
| Temperature | 25 ̊ C |
| Total number of replicate measurements | 3 |
| Applied voltage | 148 V |

Table S3. Theoretical Zn speciation calculated with Visual-MINTEQ assuming equilibrium in NCM using the exposed Zn concentrations.

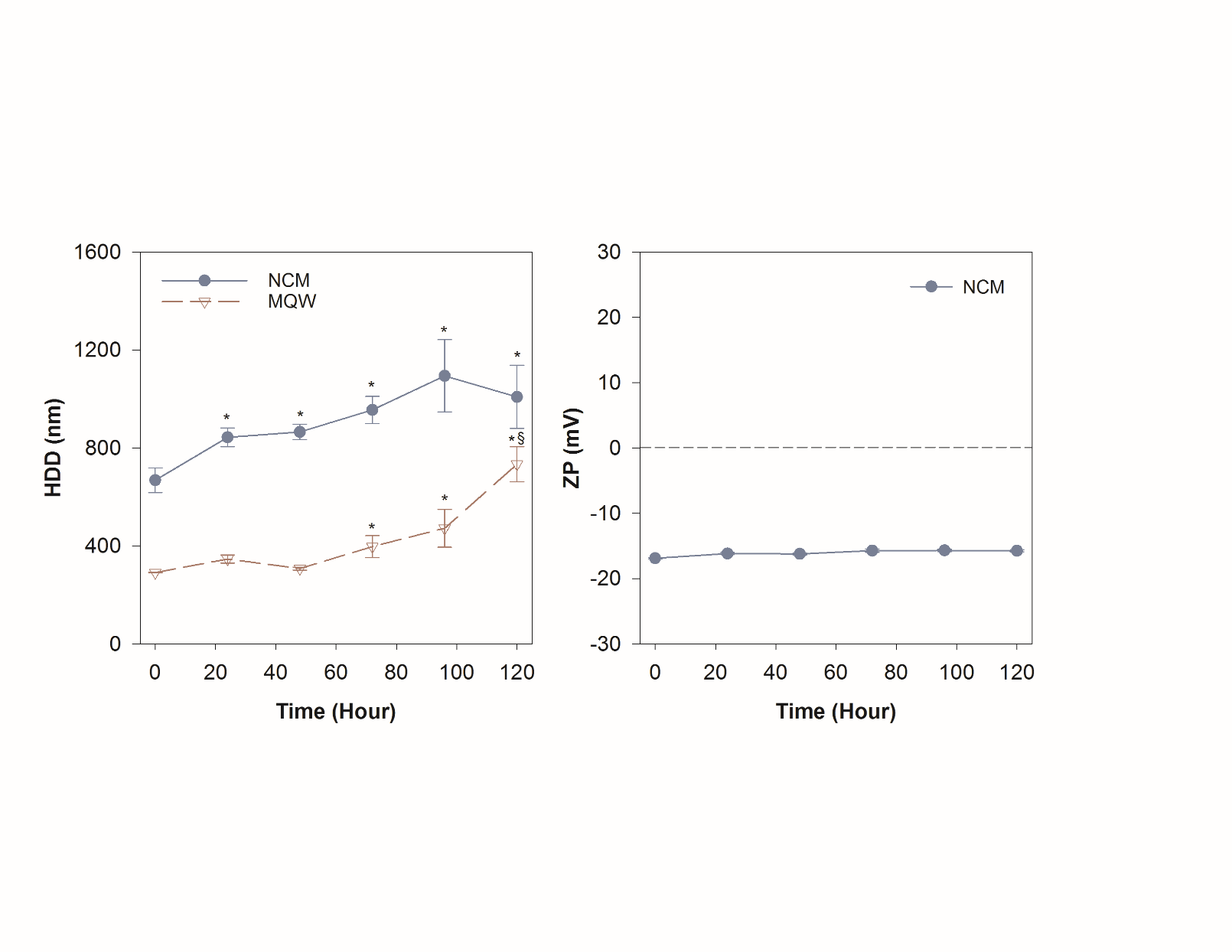


***Figure S1.*** *Zeta potential of ZnO NPs (8 mg Zn/L) in NCM over 120 hours.*


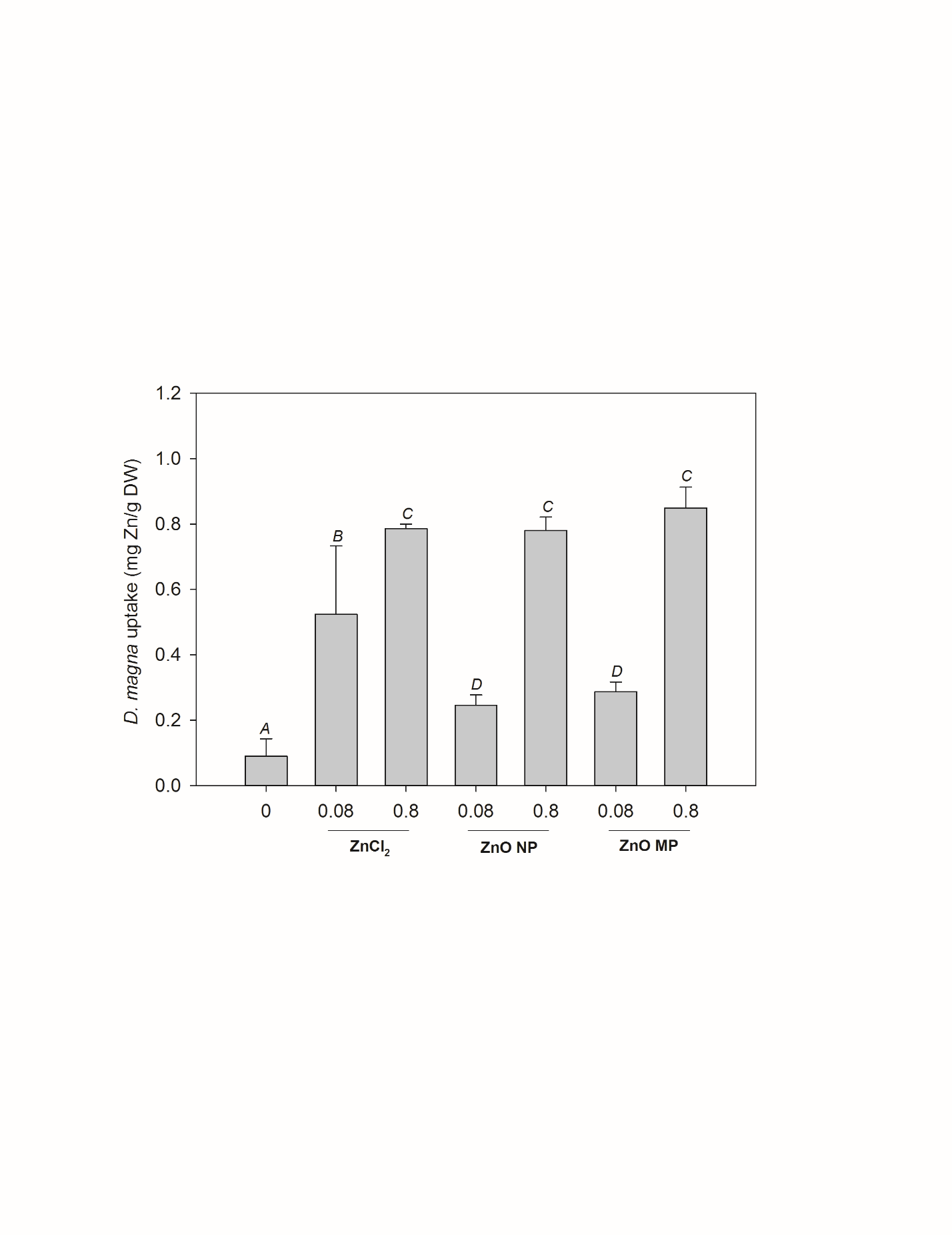


***Figure S2.*** *D. magna zinc uptake in community exposure scenario after 5 days exposure to 0.08 and 0.8 mg Zn/L. Error bars represent the standard error derived from triplicate samples. Letters indicate significant difference among exposure type and concentration.*

***Figure S3.*** *D. magna survival over 48 hours in single (a) and community (b) exposures to ZnCl_2_, ZnO NPs, and ZnO MPs. T**he asterisk (*) represents a significant difference from corresponding control,* *and symbol § indicates significant difference of ZnCl_2_ with ZnO NP and MP exposures. Error bars represent the standard error derived from triplicate samples.*

*
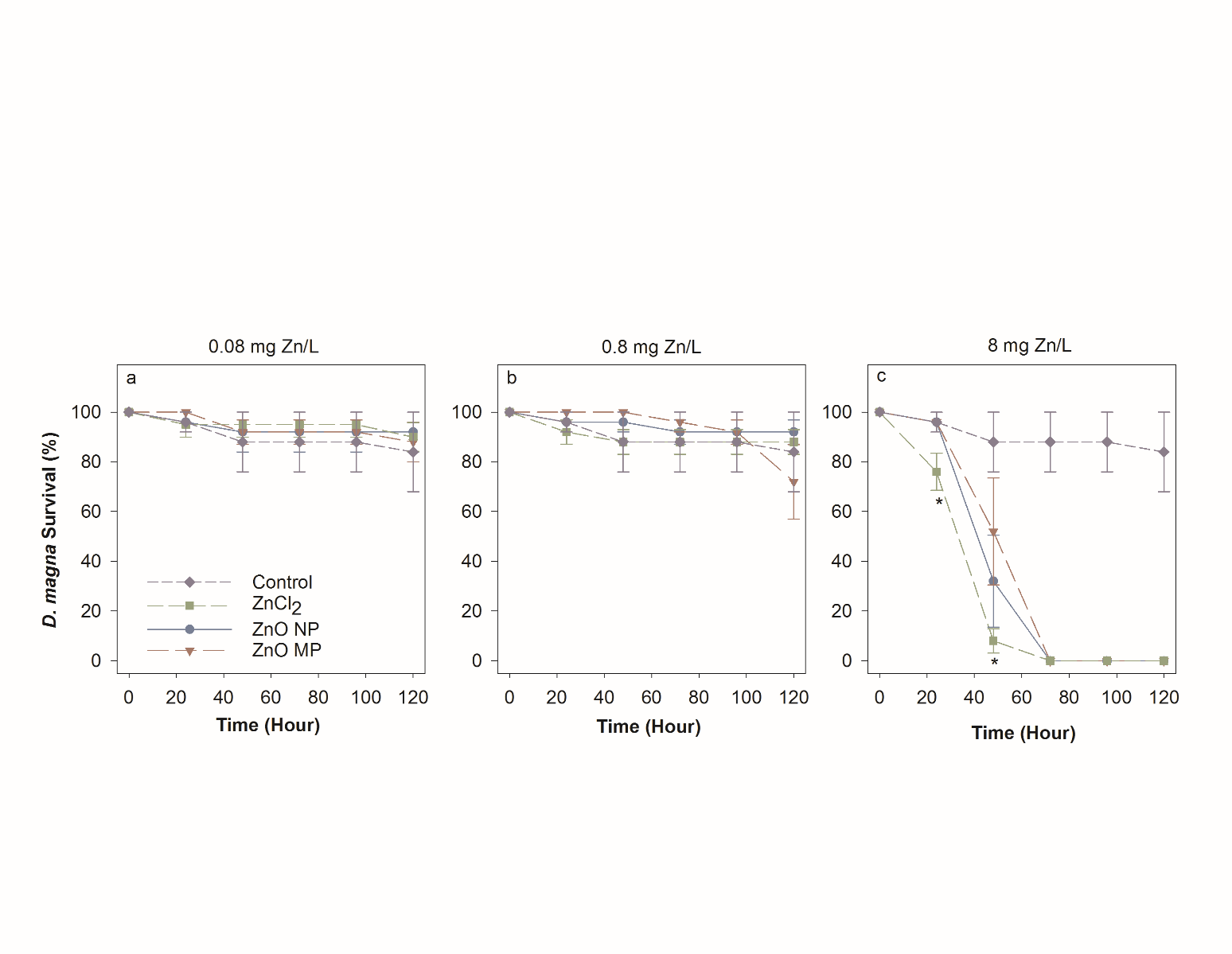
*

***Figure S4.*** *D. magna survival over 120 hours in community exposures to ZnCl_2_, ZnO NPs, and ZnO MPs at 0.08 (a), 0.8 (b), and 8 (c) mg Zn/L. The asterisk (*) represents a significant difference from corresponding control, and symbol § indicates significant difference of ZnCl_2_ with ZnO NP and MP exposures. Error bars represent the standard error derived from triplicate samples.*

*
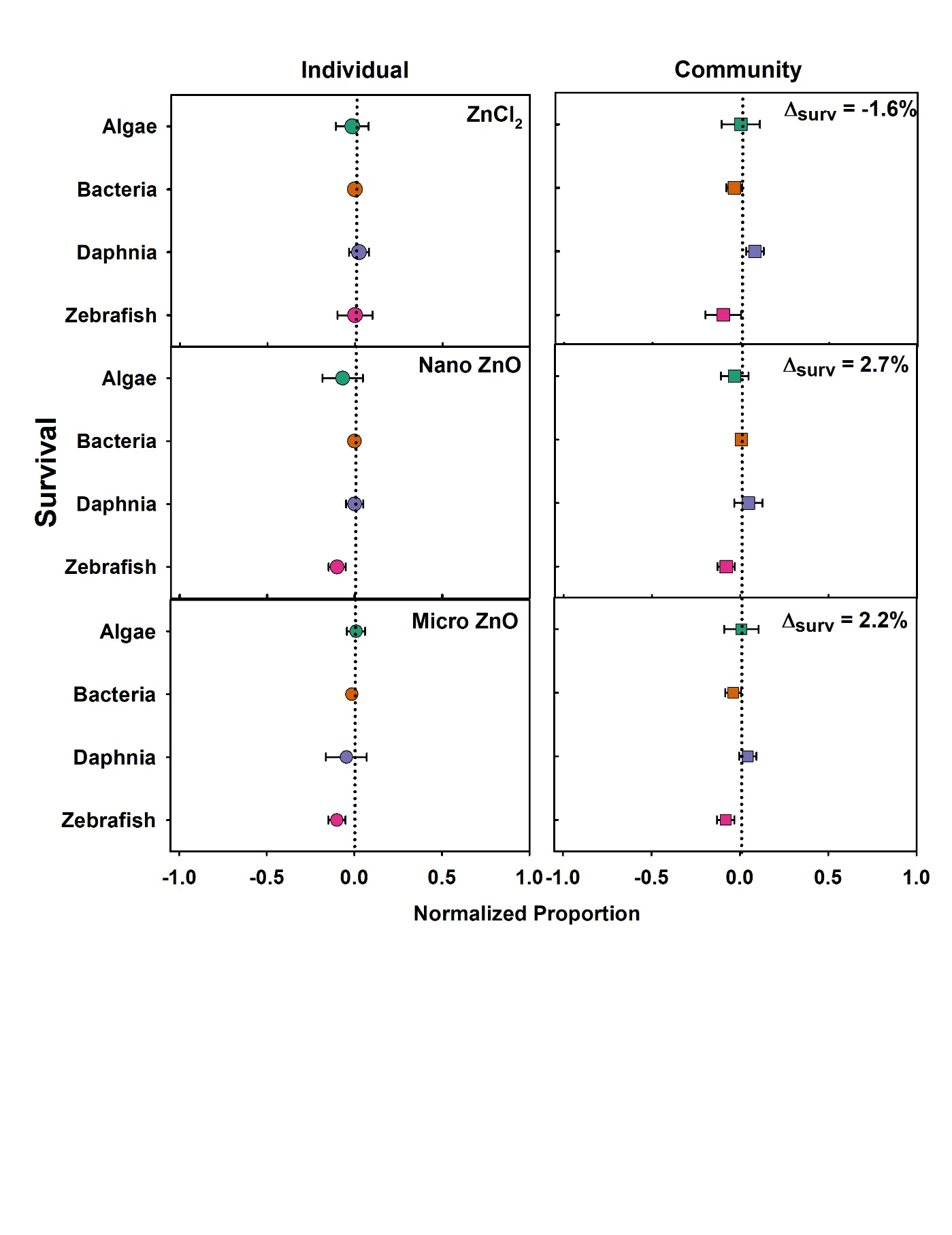
*

***Figure S5****. The overall 120-hour survival comparison for each organism in single species and NC community exposed to 0.08 mg Zn/L. The dashed line represents the normalized individual species and community control responses. Error bars represent the standard error derived from five sample replicates. The mean toxicity shift for all four species in NC community compared to single exposure scenario is represented by ∆surv.*

*
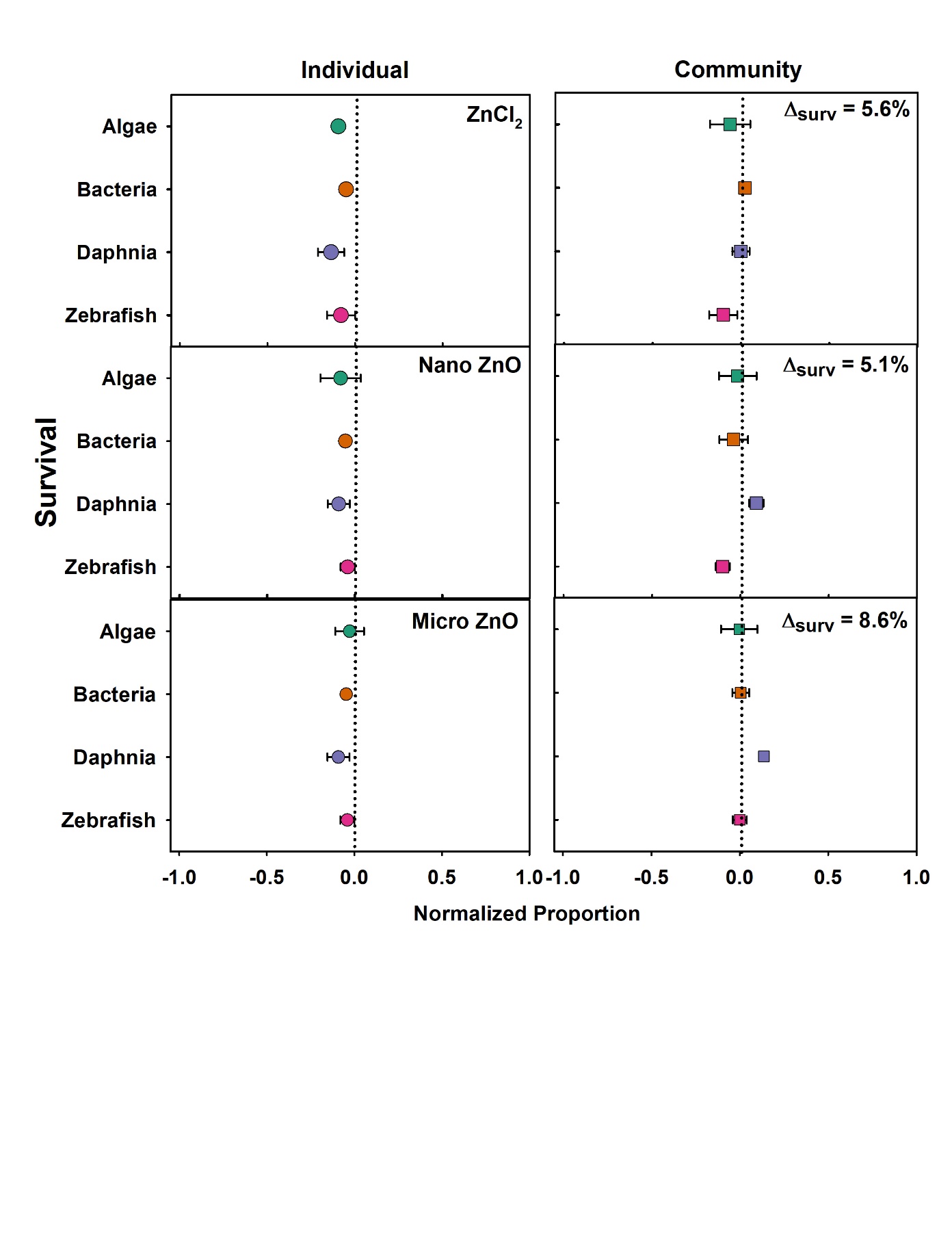
*

***Figure S6****. The overall 120-hour survival comparison for each organism in single species and NC community exposed to 0.8 mg Zn/L. The dashed line represents the normalized individual species and community control responses. Error bars represent the standard error derived from five sample replicates. The mean toxicity shift for all four species in NC community compared to single exposure scenario is represented by ∆surv.*
